# Hypoxic stress and hypoxia-inducible factors in leukemias

**DOI:** 10.3389/fonc.2022.973978

**Published:** 2022-08-18

**Authors:** Daniela Magliulo, Rosa Bernardi

**Affiliations:** Laboratory of Preclinical Models of Cancer, Division of Experimental Oncology, San Raffaele Scientific Institute, Milan, Italy

**Keywords:** hypoxic stress, bone marrow, HIF - 1α, HIF - 2, αhypoxia, inducible-factor, leukemia

## Abstract

To cope with hypoxic stress, ancient organisms have developed evolutionally conserved programs centered on hypoxia-inducible transcriptional factors (HIFs). HIFs and their regulatory proteins have evolved as rheostats to adapt cellular metabolism to atmospheric oxygen fluctuations, but the amplitude of their transcriptional programs has tremendously increased along evolution to include a wide spectrum of physiological and pathological processes. The bone marrow represents a notable example of an organ that is physiologically exposed to low oxygen levels and where basal activation of hypoxia signaling appears to be intrinsically wired within normal and neoplastic hematopoietic cells. HIF-mediated responses are mainly piloted by the oxygen-labile α subunits HIF1α and HIF2α, and current literature suggests that these genes have a functional specification that remains to be fully defined. Since their identification in the mid 90s, HIF factors have been extensively studied in solid tumors, while their implication in leukemia has lagged behind. In the last decades however, many laboratories have addressed the function of hypoxia signaling in leukemia and obtained somewhat contradictory results. Suppression of HIFs expression in different types of leukemia has unveiled common leukemia-promoting functions such as stimulation of bone marrow neoangiogenesis, maintenance of leukemia stem cells and chemoresistance. However, genetic studies are revealing that a definition of HIF factors as bona fide tumor promoters is overly simplistic, and, depending on the leukemia subtype, the specific oncogenic event, or the stage of leukemia development, activation of hypoxia-inducible genes may lead to opposite consequences. With this article we will provide an updated summary of the studies describing the regulation and function of HIF1α and HIF2α in blood malignancies, spanning from acute to chronic, lymphoid to myeloid leukemias. In discussing these data, we will attempt to provide plausible explanations to contradictory findings and point at what we believe are areas of weakness in which further investigations are urgently needed. Gaining additional knowledge into the role of hypoxia signaling in leukemia appears especially timely nowadays, as new inhibitors of HIF factors are entering the clinical arena for specific types of solid tumors but their utility for patients with leukemia is yet to be determined.

## Introduction

Oxygen supply and consumption are tightly regulated processes in multicellular organisms and when oxygen demand exceeds supply, a state of reduced oxygenation called hypoxia ensues. Hypoxia-inducible factors (HIFs) are master regulators of cellular and systemic adaptation to poor oxygen availability in physiological and pathological conditions. They promote expression of a growing number of tissue-specific genes that mediate adaptation to acute decrease in oxygen supply as well as molecular functions occurring in cell compartments that are physiologically exposed to low oxygen levels (1).

Breakthrough discoveries into the molecular mechanisms of how cells sense and adapt to oxygen availability led to the 2019 Nobel Prize in Physiology or Medicine being awarded to Drs. Gregg Semenza, William Kaelin, and Peter Ratcliffe (2).

Genes that tune cellular metabolism to atmospheric oxygen availability belong to an ancient pathway that co-evolved with oxidative phosphorylation in the first metazoans (3). Orthologues of various components of the HIF pathway (*PHD*, *FIH*, *VHL*, *HIF*, described later in detail) are conserved from simple organisms like nematodes to mammals (3) and along evolution many of these genes have been subjected to events of gene duplication. Such is the case of HIF factors, the central molecules in the hypoxia adaptation pathway, with *HIF1A* being shared by all metazoans, and *EPAS1* (the gene encoding HIF2α) and *HIF3A* having appeared at later branches of metazoan evolution. Additionally, oxygen sensing domains embedded within HIF molecules display increased molecular complexity throughout evolution, with primitive forms of HIFα expressing a single oxygen-sensitive regulatory domain and genes that have evolved later containing repetitions of these sites (3). Ongoing studies are directed to define the functional specification of the three mammalian HIFα genes in different physio/pathological cell states and cell types. However, these studies are at the very beginning, and further analyses in normal and pathological tissues will lead to a better understanding of the diversification and full regulatory potential of this pathway.

Importantly, although HIF factors have been initially defined as central regulators of cellular adaptation to acute oxygen scarcity, it must be highlighted that low oxygen levels are physiological in certain tissues or tissue microenvironments (4) and that, besides acute hypoxia, HIF factors are also expressed in cells ordinarily exposed to low oxygen, where they take part to the regulation of physiological mechanisms. Along these lines, a common semantic error is to consider the atmospheric oxygen partial pressure (pO_2_) of 160 mm Hg as normoxic, with hypoxia indicating pO_2_ values below 38 mm Hg. Although *in vitro* this is the condition where HIF factors begin to accumulate in many cell types, we must remember that most cell lines are adapted to grow at oxygen tensions as in the atmosphere, even though the tissue they originated from was probably exposed to much lower oxygen levels. Thus, the terms “hypoxia” and “normoxia” should be used attentively (5).

One typical tissue that is considered physiologically hypoxic is the bone marrow (BM), where low oxygen levels are believed to be intrinsic, especially in microenvironments devolved to the control of hematopoietic stem cells (HSCs) homeostasis (4). As such, HIF factors have been implicated in the biology of HSCs and hematopoietic cells at large and, as we will review in this manuscript, this also applies to the pathological derivatives of hematopoietic cells: leukemic cells.

## HIF pathway and regulation

Before describing the known functions and regulation of hypoxia-inducible genes in leukemia, we will use this chapter to briefly summarize the way these genes work. HIF proteins are a family of evolutionarily conserved DNA-binding transcription factors belonging to the bHLH/PAS (basic-helix-loop-helix/Per-ARNT-Sim) family. They function as heterodimers composed of an oxygen-sensitive HIFα subunit and the constitutively expressed HIF1β subunit (or aryl hydrocarbon receptor nuclear translocator, ARNT) (6). Three different HIFα genes exist in the human genome: *HIF1A*, *EPAS1* (endothelial PAS domain protein 1 gene, encoding HIF2α), and *HIF3A.* HIF1α and HIF2α bind HIF1β to assemble HIF1 and HIF2 active transcription factors, which recognize hypoxia responsive elements (5’-(A/G)CGTG-3’, HREs) in the regulatory regions of HIF-target genes (7). HIF3α is less studied than its two siblings and has been suggested to act as a dominant-negative regulator of hypoxia signaling *via* competition for HIF1β binding by some of its splicing variants and transcriptional inhibition (8). More recently, HIF3α has been identified as a lipid sensor, with endogenous lipids stabilizing its heterodimerization with HIF1β (9), which may lead to regulation of lipolysis genes in adipocytes (10). However, because information on the function of HIF3α is still scarce, in this review we will focus on HIF1α and HIF2α.

HIF proteins share homologous amino-terminal (N-terminal) domains, whilst diverging at their carboxyl-terminal (C-terminal) region. At the N-terminus, all HIF proteins carry a bHLH domain that is required for DNA binding and PAS-A and PAS-B domains that mediate heterodimerization and formation of the transcriptional complex. In the central region, an oxygen-dependent degradation domain (ODDD) is crucial for oxygen-dependent degradation of HIFα proteins as described below, and is lacking in HIF1β, thus explaining its oxygen-independent expression. At the C-terminus, HIF1α and HIF2α have two transactivation domains (N-TAD and C-TAD) that are essential to promote transcription of HIF-target genes, while HIF3α and HIF1β have only one TAD domain (11). Importantly, the C-terminal half of HIFα and HIF1β proteins contains intrinsically disordered regions (IDRs) that exert critical functions in transcriptional regulation by mediating diffusion properties and interaction with chromatin factors (12).

**Figure 1:**
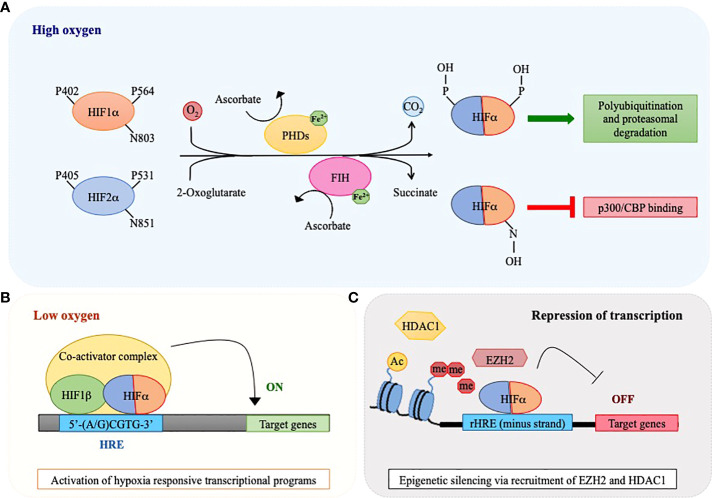
Oxygen-sensing regulatory pathway of HIF1α and HIF2α. A schematic view of HIF1α and HIF2α regulation *via* the action of oxygen sensors prolyl hydroxylases (PHDs) and factor inhibiting HIF (FIH). **(A)** PHD and FIH are HIFα hydroxylases that require iron (Fe^2+^) and ascorbate as co-factors and utilize 2-oxoglutarate and molecular oxygen as co-substrates of their enzymatic reaction, with the release of CO_2_ and succinate as waste products. In the presence of oxygen, these enzymes catalyze hydroxylation of HIF1α and HIF2α at proline (P) and asparagine (N) residues respectively, thus provoking a dual effect: PHDs cause HIFα polyubiquitination and degradation by the proteasome, while FIH inhibits binding of co-activators like CREB-binding protein (CBP) and p300 to the HIF transcriptional complex. **(B)** In conditions of oxygen scarcity, the activities of PHDs and FIH are inhibited and HIFα subunits become stabilized, dimerize with HIF1β and bind hypoxia responsive elements (HREs) within the regulatory regions of specific target genes to activate their transcription. **(C)** HIFα factors may also recognize HREs in the minus DNA strand (reverse HRE, rHRE) thus allowing recruitment of histone methyltransferases or deacetylases (EZH2 and HDAC1) and provoking epigenetic silencing and transcriptional repression.

As their name suggests, HIF proteins are importantly regulated by oxygen levels. However, HIFs are not oxygen sensors *per se*, rather they are post-translationally regulated by enzymes that use molecular oxygen to promote protein hydroxylation, thus acting as oxygen sensors (13). When oxygen is available, prolyl hydroxylases (PHD1-3) modify two conserved prolyl-residues located in the ODDD of HIFα proteins (P402/P564 in human HIF1α and P405/P531 in HIF2α) and, in so doing, promote their recognition by the von Hippel-Lindau (pVHL) E3 ubiquitin ligase that triggers their degradation by the proteasome *via* poly-ubiquitination (14, 15). In addition, an asparagine residue located in the C-TAD of HIF1α and HIF2α (N803 in human HIF1α and N851 in HIF2α) confers further oxygen-dependent regulation upon hydroxylation by the asparaginyl hydroxylase FIH (factor inhibiting HIF), which blocks interaction with the transcriptional coactivator complex p300/CBP and suppresses transcriptional activity (11, 16, 17) (**Figure 1A**). Both PHDs and FIH belong to the 2-oxoglutarate (2-OG)-dependent dioxygenase superfamily that uses oxygen and 2-OG as co-substrates together with ferrous iron (Fe^2+^) and ascorbate as obligate cofactors to catalyze protein hydroxylation (15). The activity of PHDs and FIH is inhibited under low oxygen tensions due to lack of the essential co-substrate. As a consequence, HIFα subunits are stabilized, dimerize with HIF1β and bind HREs on specific target genes together with several coactivators, including CBP/p300 (**Figure 1B**). Of note, PHD2 and PHD3 are themselves hypoxia-inducible proteins, thus providing a self-sufficient mechanism to control HIFα protein levels and ensure rapid removal of HIFα upon reoxygenation (18). Also, PHDs exhibit tissue-specific expression (for example, PHD3 is strongly expressed in the heart and PHD1 is the only isoform expressed in the testis) and differential HIFα hydroxylation, with PHD1 and PHD3 being more active on HIF2α and PHD2 hydroxylating more efficiently HIF1α (18, 19). Consequently, oxygen-dependent HIFα induction may also reflect differences in PHDs tissue distribution and activity.

Active HIF heterodimers promote transcription of an ever-growing number of genes involved in a variety of cellular functions, with important implications for both physiology and disease. Hypoxia signaling has been linked to control of cellular metabolism (with a switch from mitochondrial oxidation to glycolysis), neo-angiogenesis, regulation of cell migration and tissue invasion, regulation of cell proliferation and apoptosis, and regulation of stem cell maintenance, amongst other functions (20). Mechanistically, HIF1α and HIF2α show high sequence homology in their DNA-binding domains (21) and bind identical HRE motifs *in vitro* (7, 22), thus regulating common target genes (23, 24). However, emerging evidence indicates that HIF1α and HIF2α are endowed with important target selectivity, with HIF1α mainly promoting the expression of genes involved in glycolytic metabolism, pH regulation and apoptosis, whilst HIF2α regulates genes involved in stem cell maintenance, cell cycle and invasion (24), albeit this division of functions does not occur in all tissues and a strong element of tissue specificity is emerging in the HIF transcriptional program. The functional specification of HIFα factors has been connected to specific interactions with transcriptional coactivators occurring in their C-terminal transactivation domains, which have a lower degree of sequence homology (25). In addition, more recently it was proposed that HIFα proteins display different diffusion properties and DNA binding profiles thanks to the different amino acidic compositions of their IDRs (12). Specifically, it was revealed that HIF2α has slower nuclear diffusion than HIF1α and a tendency to bind negatively charged chromatin-associated RNA and proteins due to the preeminent presence of positively charged amino acids in its IDR (12). Adding to these intrinsic properties, tissue- and developmental-specific expression and different sensitivity to oxygen levels lead to an emerging pattern of differential gene regulation by HIF1α and HIF2α (23, 24, 26).

Interestingly, it has been suggested that the diversification of HIFα functions may originate from their evolutionary history (3). Having evolved to adapt cellular physiology to acute oxygen scarcity, *HIF1A*, which is the most ancient member of the family, is presumed to be the primary regulator of metabolic rewiring, while the later appearance of *EPAS1* may have led to a wider spectrum of functions, including processes that occur within cell types residing physiologically at low oxygen levels (i.e. stem cells). However, as we will describe later, such a clear diversification of functions may not apply to all tissues or cells.

In conclusion, it is worth mentioning that, beside their role as transcriptional activators, HIFα have also been described as transcriptional repressors for certain genes (e.g. peroxisome proliferator activated receptor (PPAR)α, α-fetoprotein, leukemia inhibitory factor receptor) *via* binding to HREs located on the minus DNA strand (also called reverse HREs, rHREs) (27–29). Recently, binding of HIFα subunits to rHREs has been shown to repress transcription *via* epigenetic silencing promoted by recruitment of histone modifiers like HDAC1 and EZH2 (the catalytic subunit of Polycomb repressive complex 2, PRC2) (30–32) (**Figure 1C**). Because HIF factors also promote the expression of a number of epigenetic factors, epigenetic mechanisms are emerging as a relevant way of controlling gene expression downstream hypoxia signaling (33).

## Low oxygen tensions in healthy bone marrow and in leukemia

In humans, oxygen gradients differ greatly among tissues and tissue microenvironments. In the BM, oxygen levels are overall lower than in many other organs, and hematopoietic cells experience physiological pO_2_ ranging from 32 mm Hg in arteriole-rich endosteal zones to 9.9 mm Hg in deeper regions (34, 35). The BM is home to a variety of non-hematopoietic cell types that constitute specialized microenvironments, known as niches, where stem cells, progenitors and more differentiated hematopoietic cells reside and are physiologically regulated (36). Despite its extensive vascularization, the BM is considered a tissue low in O_2_, likely due to the extent of oxygen consumption caused by its high cellularity. Initial studies using in silico modeling of oxygen BM gradients and quantification of BM perfusion led to the assumption that HSCs reside near the poorly perfused endosteal niche and show signs of hypoxia due to their distance from oxygen-rich blood vessels (37–39). Despite this general assumption, measuring the exact oxygen tension and physical location of niches low in O_2_ in the BM is technically challenging, therefore indirect measurements have been often utilized, such as evaluation of HIF1α expression or incorporation of hypoxia markers like pimonidazole (Pimo) and Hoechst 33342 (35, 38, 39). However, use of chemical agents, such as Pimo and Hoechst 33342 may be misleading as they do not provide a direct assessment of O_2_ levels and may rather offer a readout of specific cellular conditions. As an example, Pimo, which competes with oxygen as an electron acceptor, may display increased reductive activation not only as a consequence of decreased intracellular oxygen, but also upon distinctive metabolic shifts from mitochondrial to glycolytic catabolism (40). Similarly, uptake of the fluorescent DNA intercalant Hoechst 33342, which has been used to measure blood perfusion as an indirect marker of tissue oxygenation, may vary depending on cell proliferation rates. Conversely, use of two-photon phosphorescence lifetime microscopy, which directly measures oxygen levels, revealed that the BM is overall a tissue low in O_2_ where intravascular oxygen tensions are quite similar in endosteal and sinusoidal vessels (21.9 and 17.7 mm Hg respectively), and a moderate extravascular oxygen gradient exists, with higher oxygen levels in the endosteal region and decreased oxygen towards the sinusoidal region (34). Of note, these experiments have been performed in the calvaria, which may not represent all BM compartments, as Pimo staining revealed that hypoxic cells are less frequent in this site compared to long bones (41). Therefore, it remains unclear whether the current knowledge on BM oxygen levels may be relevant for most bones.

Nonetheless, the notion that hematopoietic cells thrive in an environment that is low in O_2_ has been validated over the years by a number of *in vitro* or *ex vivo* experiments demonstrating that low oxygen levels: i) promote HSCs quiescence and maintenance (42–48), while at the same time supporting a balanced differentiation program towards many hematopoietic lineages (49–52); ii) favor a metabolic switch to (anaerobic) glycolysis thus exerting protection from oxidative stress that may cause DNA damage (35). Also, hypoxia efficiently enhances *in vitro* expansion and *in vivo* engraftment of HSCs (53–55).

More recently, it was revealed that, besides external oxygen availability, HSCs display an inherent hypoxic state that is independent of their exact location within the BM and may be rather dictated by internal metabolic activity (40). In this respect, it must be emphasized that expression and activity of HIF factors is not only regulated by oxygen levels but also by a variety of oxygen-independent mechanisms that promote their expression and transcriptional activity. Providing a list of these mechanisms is beyond the scope of this review, as it has been discussed elsewhere (56, 57), but suffice it to say that they include extracellular stimuli like growth factors, cytokines and chemokines. Thus, the molecular milieu of different BM niches may participate considerably to the regulation of hypoxia signaling within hematopoietic cells. Whatever the triggering event, one certain finding is that HIF factors are highly expressed in hematopoietic stem and progenitor cells (HSPCs), where they play important functions. This was clearly demonstrated in knock-out mice, where it was shown that HIF1α exerts a critical cell-autonomous functions in promoting HSCs quiescence (58), and HIF2α promotes HSCs maintenance predominantly *via* regulation of the BM microenvironment (59).

In the case of hematological malignancies, leukemic cells are great oxygen consumers, but at the same time promote BM neoangiogenesis, which increases nutrients and oxygen availability (60). Thus, it is not clear if abundance of proliferating leukemic cells and high oxygen and nutrient consumption may further decrease oxygen availability in the leukemic BM. By measuring BM blood gas levels in healthy volunteers and AML patients, two independent reports observed similar pO_2_ of around 47 mm Hg (61, 62). Conversely, *in vivo* intracellular hypoxia labeling with the indirect marker 2-nitroimidazole in a rat model of AML showed increased reactivity during disease progression and in comparison with healthy animals (63). More recently, direct measurement of oxygen tension in leukemic BM was obtained in a BCR-ABL B-ALL mouse model by intravital fast scanning two-photon phosphorescence lifetime imaging microscopy, which showed that oxygen levels vary from the initial to the final stages of leukemia, with a transient increase in oxygen levels at intermediate leukemia burden, correlating with expansion of the vasculature network, and a later significant decrease of BM oxygenation as leukemia cellularity increased at disease end-stage (64). By suggesting that different oxygen levels characterize different stages of leukemia development, these findings may provide an explanation to the apparently contradictory findings described above. However, as previously discussed for normal BM, also and particularly in a leukemic BM where the cytokine milieu is perturbed towards pro-inflammatory and leukemia-sustaining cytokines, intracellular hypoxic pathways may be activated *via* additional routes. In this respect, work from our and other laboratories has shown that HIF1α is transcriptionally upregulated when leukemic cells are cocultured with BM mesenchymal cells (65–67). Also, a number of studies that we will describe in the next sections have reported that upregulation of HIF factors in leukemic blasts does not necessarily occur at the post-translational level, thus implying oxygen-independent mechanisms. In the following chapter, we will provide a summary of the regulation and function of HIF1α and HIF2α across blood malignancies.

## HIFs and hypoxia signaling in leukemia

Since their cloning and molecular characterization, the function of HIF factors has been intensely investigated in solid tumors. More recently, increasing research efforts have also focused on the involvement of hypoxia signaling in leukemia, albeit with somewhat controversial results that fail to provide a unanimous consensus. Depending on the type/subtype of leukemia, or the experimental approach that has been utilized (knock-out animals versus knock-down experiments in cell lines/primary cells), HIFs have been described as tumor suppressors, oncogenes, or neutral genes even within the same leukemia. In the next paragraphs, we will summarize current knowledge on the expression and activity of HIF factors in different leukemic contexts (acute and chronic, myeloid and lymphoid) and discuss possible explanations to controversial evidence recently published. Of note, the majority of the studies that we will describe have focused on defining the functions of HIF1α, whereas HIF2α has been much less investigated and detailed analyses on the functions of this factor in leukemia establishment and progression are still lacking.

### Expression of HIFα genes and proteins in leukemia

Expression of HIFα factors in leukemia has been extensively studied at both mRNA and protein levels and increased expression of HIFα subunits has been found in various blood malignancies compared to normal cells (**Table 1**) (77). One of the first reports of HIF1α upregulation in leukemia came from studies in chronic myeloid leukemia (CML), where it was observed that BCR-ABL induces HIF1α mRNA and protein expression downstream PI3K/mTOR activation (68). Later on, this finding was confirmed in primary CML cells where the HIF1α transcript was found more expressed in leukemic cells compared to BM from healthy volunteers (69). In acute myeloid leukemia (AML), a disease characterized by broad genetic and morphological heterogeneity, a general increase in HIF1α and HIF2α protein levels was reported in mouse and human AML cells when compared to normal BM leukocytes (70). In addition, expression of HIFα transcripts was reported to vary in morphological and molecular subtypes, and this may echo functional cooperation with specific oncoproteins. For example, HIF1α mRNA was found particularly elevated in AML with the t(8;21) translocation (encoding RUNX1-RUNX1T1, or AML1-ETO), where it associates with unfavorable prognosis and tumor aggressiveness (71). Functionally, HIF1α and AML1-ETO were found to engage in a positive regulatory circuit where they stimulate their reciprocal expression and cooperate to alter DNMT3a levels and global DNA methylation towards increased AML proliferation (71). In another study, HIF2α expression was found elevated in M3 and M6 AMLs, as defined by FAB morphological classification, and in AMLs with t(15;17) translocation (generating the PML-RARα oncoprotein), inv(16) and complex karyotype (70), although a functional cooperation of HIF2α with the oncogenic drivers of these AML subtypes was not tested.

**Table 1:** Expression of HIF1α and HIF2α in leukemia.

Leukemia	HIF1α	HIF2α	Site of detection	References
CML	mRNA, protein	–	Murine cell line	(68)
	mRNA	–	Normal and CML BM samples	(69)
AML	protein	mRNA protein	Normal and AML mouse BM samples (HIF1α), human primary cells and cell lines (HIF2α)	(70)
*with AML1-ETO*	mRNA	–	Normal and AML BM samples, human cell lines	(71)
CLL	mRNA protein	–	Normal and CLL BM and PB samples	(65, 72–74)
*with TP53^mut^*	mRNA protein	–	Normal and CLL PB samples, human cell lines	(66)
ALL	protein	mRNA, protein	Normal and ALL mouse BM samples (HIF1/2α), human cell lines (HIF2α)	(70)
	protein	–	ALL BM samples	(75, 76)

CML, chronic myeloid leukemia; AML, acute myeloid leukemia; CLL, chronic lymphocytic leukemia; ALL, acute lymphoblastic leukemia; BM, bone marrow; PB, peripheral blood.

In addition to these studies, recent work has reported that HIF1α-target genes are upregulated in AML cells carrying *TP53* mutations, although HIF1α levels were not evaluated (78). A connection between HIF1α and mutant p53 was reported also in chronic lymphocytic leukemia (CLL). In this disease, it was first observed that HIF1α is highly expressed at the mRNA and protein level compared to normal B cells (72–74), which correlates with leukemia progression (79). In addition, more recently it was reported that HIF1α expression is higher in patients with *TP53* mutations compared to wild-type *TP53* (66). Taken together, these data suggest that a connection between *TP53* mutational status and HIF1α expression and/or function exists in different leukemic contexts. However, it remains to be elucidated whether HIF1α specifically cooperates with gain of function *TP53* mutants in leukemia, as it has been established in some solid tumors and lymphoma (80).

Of note, constitutive expression of the HIF1α protein in CLL cells is also driven by post-translational stabilization due to miR-92-1-mediated pVHL downregulation (73), thus indicating that multiple mechanisms converge into elevating HIF1α expression in this disease. Although HIF2α expression has not been measured in CLL cells, some of these mechanisms may also promote HIF2α upregulation (e.g. pVHL suppression).

In acute lymphoblastic leukemia (ALL), HIF1α expression has been measured *via* immunostaining of BM biopsies, which revealed that HIF1α is overexpressed in BM of childhood ALL (75) and correlates with worse overall survival (76). In addition, HIF2α protein expression was found increased in a subset of primary ALL cells compared to normal hematopoietic cells (70).

Besides increased basal expression of HIF factors compared to normal cells across different leukemias, as mentioned above, expression of HIF1α within the CLL and ALL leukemic compartment increases upon co-culture of leukemic blasts with BM stromal cells (65, 66, 76). Consistently, transcriptomic analyses of primary CLL cells cultured on human stromal cells revealed that hypoxic signatures are amongst the most upregulated (81). A crosstalk between the BM microenvironment and leukemic cells towards increased hypoxia signaling was also found in AML, where recent evidence obtained in *ex vivo* and *in vivo* models demonstrated that AML cells impair normal hematopoiesis by rewiring the transcriptome of mesenchymal stromal cells *via* increased HIF1α expression (82).

In conclusion, current literature indicates that upregulation of HIFα factors and their target genes may be a general phenomenon in leukemia with respect to normal hematopoietic tissue, occasionally with further accumulation in specific genetic backgrounds or upon not yet fully characterized environmental cues provided by BM microenvironments. Of note, many of these studies describe upregulation of HIFα factors both at the mRNA and protein levels, thus revealing that increased HIFs expression in leukemia is not only caused by hypoxic post-translational stabilization.

A note of caution in the interpretation of these studies is that whole BM hematopoietic tissue has often been used as the normal counterpart of leukemic cells, although BM aspirates contain a variety of cell types that are much more heterogeneous than leukemic blasts. Thus, defining the real extent of HIFs overexpression in leukemia remains an open question. Nonetheless, functional studies have provided strong evidence of the involvement of hypoxia signaling in leukemogenesis and drug resistance. These will be described next, along with contradictory studies suggesting that HIF factors may be endowed with tumor-suppressive functions in some leukemias.

### HIF1α and HIF2α support leukemia maintenance and propagation

**Figure 2:**
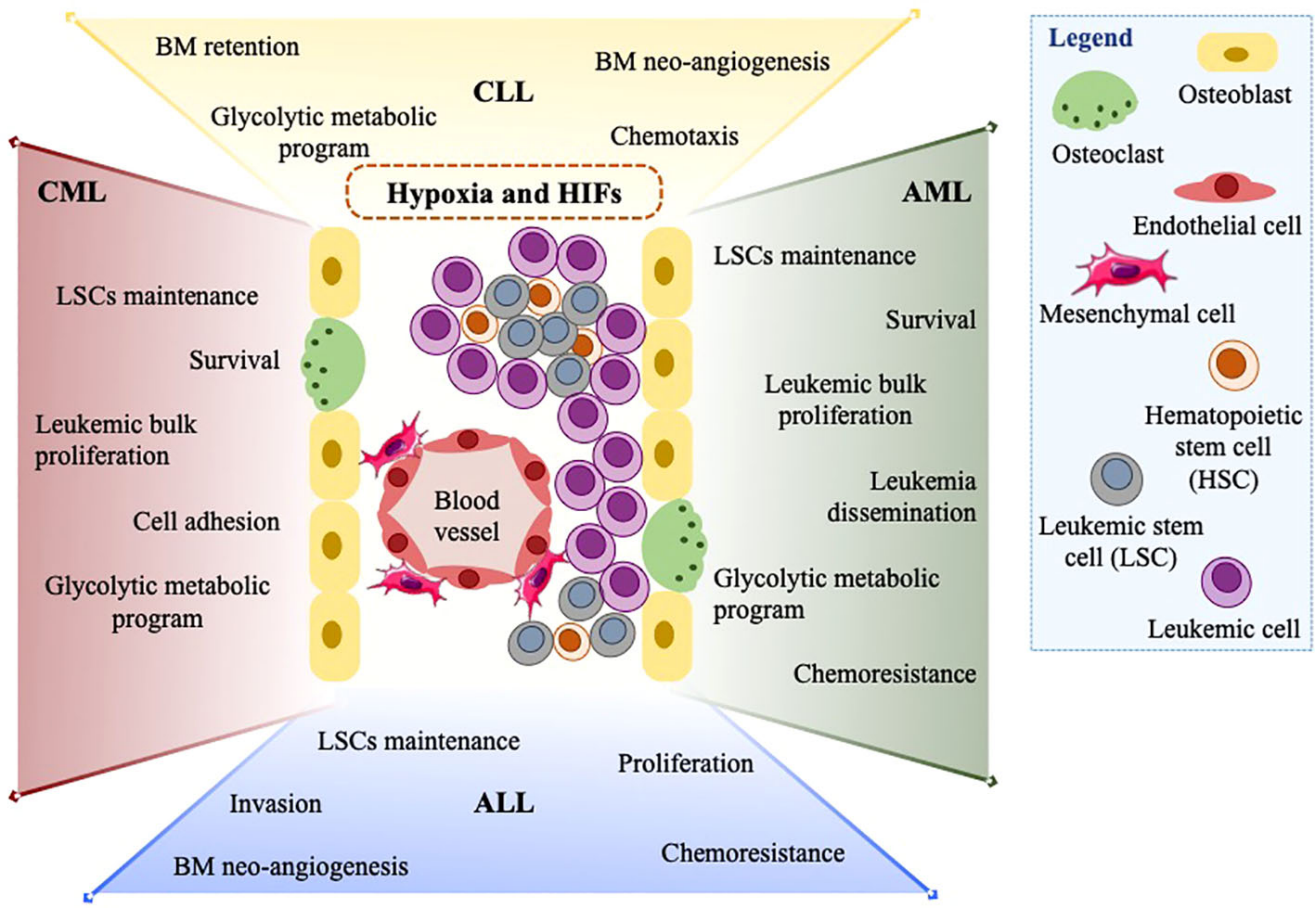
The assortment of HIF1α and HIF2α functions in leukemias. In the leukemic BM microenvironment, high cellularity and oxygen consumption expose leukemic cells to hypoxia and promote the expression of HIF1α and HIF2α. HIFs and hypoxia exert a wide range of tumor-promoting functions in many leukemia types (CML, CLL, ALL, and AML). Amongst others: maintenance of LSCs, increased leukemic cell proliferation, metabolic switch to glycolysis, and chemoresistance are summarized in this figure.

In the last decades, several studies have provided evidence that HIFα factors exert relevant tumor-promoting functions in leukemia (**Figure 2**). At first, hypoxia-regulated genes were implicated in fostering VEGF production and BM neo-angiogenesis in CLL and ALL (73, 75). Following these reports, HIFα factors, prevalently HIF1α, have been attributed other important tumor-promoting functions, with some leukemia-specific nuances.

Perhaps the most concordant role of HIF1α across diverse types of leukemia is to promote maintenance of leukemia stem cells (LSCs), a function that HIF1α also exerts in the non-malignant hematopoietic stem cell compartment (58). Different groups have shown that hypoxia conditioning prompts decreased proliferation and longer maintenance of leukemia initiating cells in primary CML and AML (83, 84). Microarray analysis of CD34^+^ CML progenitor cells exposed to hypoxia revealed increased expression of cell adhesion and survival genes, concordantly with decreased apoptosis and improved colony-forming potential, thereby suggesting that targeting HIF1α along with BCR-ABL may represent a therapeutic opportunity for eradication of LSCs in CML patients (85). The crucial role of HIF1α in promoting maintenance of CML LSCs was also validated in a BCR-ABL transgenic mouse model, where HIF1α conditional deletion resulted in impaired LSCs propagation caused by delayed proliferation and induction of apoptosis *via* expression of p16^Ink4a^, p19^Arf^ and p53 (86). In AML, implication of HIF1α in LSCs maintenance was first described *via* activation of Notch1 and expression of Hes1, a transcription factor with essential stem cell-promoting roles (87). In addition, a context-specific function of HIF1α was described in AML cells carrying the AML1-ETO oncoprotein, where HIF1α sustains LSCs maintenance by transcriptional cooperation with AML1-ETO (71). In line with these data, it was recently suggested that inhibiting HIF1α with compounds like echinomycin may represent a new therapeutic opportunity to impair maintenance of the LSCs population, and this may be particularly relevant in the genetic background of *TP53*-mutated AMLs, where echinomycin showed a potent cytotoxic effect and affected leukemia propagation in xenograft mouse models (78). Similarly, the HIF1α inhibitor acriflavine impaired the stem cell potential of primary CML cells and murine BM cells transduced with *BCR-ABL*, thus suggesting that acriflavine may add therapeutic value to currently used tyrosine kinase inhibitors that target BCR-ABL by preventing CML relapse (88).

Besides promoting maintenance of a subpopulation of leukemic cells characterized by specific surface markers or functional properties (LSCs), HIF signaling also drives proliferation of bulk leukemic cells in CML and AML. For example, knockdown of HIF1α led to a marked reduction in cell proliferation in the CML cell line K562 (69) and hypoxia conditioning promoted proliferation of AML KG-1 cells (89). Also, pharmacologic inhibition of HIF1α in AML cells carrying the AML1-ETO or PML-RARα oncoproteins suppressed leukemia expansion *in vivo* (71, 90).

A dual activity of HIF1α in supporting proliferation of leukemia bulk and maintenance of leukemia-repopulating cells was also observed in T-ALL. Mechanistically, it was demonstrated that HIF1α activates Notch1 signaling and T-ALL cell proliferation (91), while also promoting Wnt signaling *via* upregulation of β-catenin, thus supporting LSCs maintenance (92).

In T-ALL, HIF1α-mediated Notch1 activation was also implicated in promoting leukemia cell invasion *via* expression of metalloproteases MMP2 and MMP9 (91). Interestingly, a similar role of stimulating leukemia dissemination was reported in AML sub-types including acute promyelocytic and monocytic leukemia, where HIF1α induces chemokine-dependent cell migration and transcriptional programs of epithelial to mesenchymal transition (90, 93, 94). Consistently, in the AML cell line KG-1, hypoxia conditioning promotes epithelial-mesenchymal transition *via* activation of PI3K/Akt (95).

In summary, across different types of leukemia, HIF1α has been implicated in the regulation of LSCs maintenance as well as in promoting leukemia dissemination, at times *via* transcriptional programs reminiscent of epithelial to mesenchymal transition in neoplastic epithelial cells. These results are in line with a recognized link between epithelial to mesenchymal transition programs and formation of cancer stem cells in solid tumors (96), and suggest that a similar connection may also occur in hematopoietic malignancies *via* activation of HIF factors within other mechanisms.

In apparent contrast to these findings, in CLL HIF1α regulates the expression of genes involved in cell adhesion and BM homing, with the ultimate effect of promoting BM retention and chemoresistance (65). These studies highlight the diversity of HIF-dependent transcriptional programs and cellular functions in different leukemic contexts, a concept that is beginning to be widely appreciated and is probably due in large part to chromatin accessibility and tissue-specific epigenetic landscapes (33).

Another function of HIF factors that appears conserved in distinct leukemias and is in line with their ancestral evolutionary function is modulation of metabolic activity. In CLL, hypoxia conditioning causes a metabolic shift from mitochondrial metabolism to glycolysis that occurs *via* activation of classical HIF-target genes (65, 97, 98). Interestingly, in AML and CML cell lines, as well as normal hematopoietic progenitor cells, it was reported that both HIF1α and HIF2α promote the expression of genes belonging to the glycolytic program (99). Surprisingly, knock out of either gene or their obligate heterodimeric partner HIF1β did not result in impaired glucose consumption and lactate production, albeit abolishing expression of glycolytic genes. Accordingly, the proliferation capacity of K562 cells was not impacted by HIF-1α, HIF-2α or HIF-1β knock-out (99). This study suggests that the oncogenic properties of HIF genes in leukemia may not rely on their metabolic functions or that adaptive metabolic rewiring compensates for deficiency in hypoxia signaling. In addition, this work provides evidence of a strong contribution of HIF2α in regulating the expression of glycolytic genes, albeit this function may be tissue-specific.

Few other studies have investigated the role of HIF2α in leukemia, and they focused specifically on AML. Silencing of HIF2α in primary AML cells cultured *ex vivo* resulted in impaired proliferation and reduced engraftment in recipient mice. Mechanistically, this phenotype was linked to HIF2α-mediated protection from apoptosis induced by ER stress and UPR response (100). In line with these results, ectopic expression of HIF2α accelerated leukemia progression in mice, while its knockdown in a human AML cell line reduced proliferation and prolonged survival of transplanted mice (70). As of today, the function of HIF2α in other types of leukemia has not been reported.

### Implication of HIF factors in drug resistance

Hypoxia and HIFα factors have been implicated in mechanisms of resistance to chemotherapeutic agents in different leukemias. In B-ALL, hypoxia conditioning was found to increase expression of anti-apoptotic genes thus dampening the effect of chemotherapeutic compounds (101). Similarly, low oxygen levels lowered T-ALL cell sensitivity to chemotherapy and preserved their ability to initiate leukemia progression *in vivo*, while silencing of HIF1α sensitized leukemic cells to treatment, thus pointing to HIF1α as an important regulator of T-ALL chemoresistance (102). In line with these data, activation of Notch1 by HIF1α also resulted in protection of leukemic T-ALL cells from dexamethasone-induced apoptosis (91).

Similar results have been reported for AML, albeit *via* different molecular mechanisms. Hypoxic exposure of AML LSCs promotes chemoresistance to cytarabine arabinoside (Ara-C) *via* upregulation of the Polycomb transcriptional repressors BMI-1, which in turn supports malignancy *via* activation of PI3K/Akt signaling and EMT programs (95). The influence of hypoxia exposure on Ara-C susceptibility *via* HIF1α expression was also confirmed in a panel of leukemic cell lines, and this was suggested to represent a possible mechanism of minimal residual disease maintenance in the bone marrow after chemotherapy (103).

Besides promoting resistance to general chemotherapeutic drugs, hypoxia signaling has also been implicated in resistance to targeted therapy, specifically to the BCR-ABL tyrosine kinase inhibitor imatinib (104). Imatinib has become the main therapeutic opportunity for CML patients, but resistance often occurs even after prolonged treatment exposure (105). Interestingly, imatinib-resistant CML cells were shown to exhibit non-hypoxic upregulation of HIF1α and its target genes, resulting in upregulation of glycolytic processes, increased glucose uptake and lactate production (106). Recently, it was suggested that activation of glycolytic metabolism in CML cells may be facilitated by reduced expression of miR-18a-5p, which targets the 3’-UTR of HIF1α and leads to HIF1α downregulation in normal hematopoietic cells (107). In line with a relevant function of HIF1α in resistance to imatinib, it was shown that although imatinib partly inhibits HIF1α expression and transcriptional activity, HIF1α residual function is sufficient to suppress imatinib-induced apoptosis of CML cells (85). This may be particularly true in bone marrow microenvironments low in O_2_, where hypoxia signaling may effectively overcome BCR-ABL inhibition.

In conclusion, hypoxia and activation of HIF signaling take an important part to resistance mechanisms to chemotherapeutic agents and targeted therapy in different types of leukemia. As a consequence, a combination of chemotherapy and compounds targeting the hypoxic pathway may represent a valuable therapeutic approach for some types of leukemia. Along these lines, we and others have recently demonstrated that in CLL targeting HIF1α with different compounds increases response to current CLL therapeutic strategies including fludarabine and ibrutinib (66, 108). In more detail, HIF1α inhibition with BAY87-2243 causes downregulation of the HIF1α targets CXCL12 and CXCR4 thus abrogating the pro-survival effect exerted by stromal cells and promoting fludarabine-induced apoptosis (66, 67). Concordantly, inhibition of HIF1α with the camptothecin derivative EZN-2208 exerts anti-tumor activities and acts as a chemosensitizer by interrupting protective microenvironmental interactions of CLL cells both *in vitro* and *in vivo* (108). Therefore, we posit that this may be an interesting direction for future investigations into improving leukemia treatment, also in view of the availability of novel small molecule inhibitors of HIF2α (109), whose function should be investigated in leukemia.

### HIFα factors as tumor suppressors in leukemia

As stated at the beginning of this chapter, studies on the role of HIF1α and HIF2α in leukemia failed to produce universal consent, especially in AML and CLL where contrasting evidence has been reported (**Table 2**). This is particularly evident in AML, where the function of HIFα factors has been studied by various groups. Beside the implication of HIF1α and HIF2α in LSCs maintenance, leukemia proliferation and survival that was previously described, other studies have revealed that HIF1α and HIF2α may display tumor-suppressive roles or no relevant functions in AML development and progression. For instance, deletion of the HIF1α gene in mouse hematopoietic stem and progenitor cells alongside retroviral transfer of AML oncogenic drivers (MLL-AF9, AML1-ETO, or MEIS1 and HOXA9) failed to impact leukemia initiation, progression and LSCs self-renewal by serial transplantation experiments (111). Rather, in the case of MLL-AF9-driven leukemia, HIF1α deletion accelerated leukemia progression by increasing cell proliferation (111). In addition, inducible HIF1α deletion in the MLL-AF9 model resulted in increased recovery upon withdrawal of chemotherapeutic regimens (112), thus suggesting that HIF1α inhibition cannot be expected to improve chemotherapy sensitivity in all leukemia. Of note, in the AML1-ETO mouse model it was reported that genetic deletion of the HIF1α gene resulted in compensatory expression of HIF2α (111), which led the authors to suggest that HIF2α upregulation may lead to increased leukemia aggressiveness, at least in some genetic backgrounds of AML. However, other authors reported that HIF2α gene deletion in MEIS1/HOXA9 and MLL-AF9 mouse models also shortened AML latency and seemed dispensable for LSCs maintenance, an effect that was even potentiated by HIF1α co-deletion (113).

**Table 2:** Contrasting reports on HIFα functions in leukemia.

Leukemia	Cell/Mouse model	Phenotype	References
CLL	HIF1α knock-out in Eμ-TCL1 mouse model	No impact on leukemia progression and survival	(110)
	HIF1α inhibition by BAY87-2243 or EZN-2208 in primary cells	Impairment of protective microenvironmental cues Chemosensitization	(67, 108)
AML	HIF1α inhibitor in primary cells	Maintenance of LSCs	(87)
	HIF1α shRNA and inhibitors in human and murine cell lines *in vitro* and *in vivo*	Disease progression by cooperation with oncogenic fusion proteins (AML1-ETO and PML-RARα)	(71, 90)
	HIF2α shRNA in primary cells	Protection of LSCs from apoptosis induced by reactive oxygen species	(100)
	HIF2α ectopic expression and shRNA in primary cells	Increased leukemia progression	(70)
	HIF1α knock-out in AML1-ETO9a and MEIS1/HOXA9 mouse models	No impact on leukemia establishment, progression and LSCs maintenance	(111)
	HIF1α knock-out MLL-AF9 mouse model	Increased leukemia progression	(111, 112)
	HIF2α sgRNA-Cas9 in THP1 cell line	No impact on cell survival, proliferation and colony formation	(113)
	HIF2α knock-out in MEIS1/HOXA9 and MLL-AF9 mouse models	Accelerated leukemia progression and reduced mice survival No effects on LSCs maintenance in secondary transplantations	(113)

Similar head-scratching results have been recently published for CLL, where it was shown that knock-out of the HIF1α gene in the Eμ-TCL1 mouse model did not impact CLL progression or increase mice survival, suggesting that HIF1α is not essential for CLL leukemogenesis (110). This genetic experiment is particularly puzzling in view of the significant upregulation of hypoxic gene signatures that was reported in leukemic cells from Eμ-TCL1 mice (110), thus confirming data obtained by other laboratories of augmented hypoxia signaling in CLL (65–67). However, HIF1α knock-out did not affect the transcriptional program of Eμ-TCL1 leukemic cells (110). Therefore, the authors of this paper speculated that compensatory mechanisms may occur that render HIF1α inactivation of no consequence. Alternatively, dependency on HIF factors may be triggered by specific leukemogenic mutations and not others, an explanation that may very well apply also to AML.

These explanations are equally plausible, but we would like to argue that another possible explanation is that HIF factors exert different functions at distinct stages of leukemia development and progression. Thus, genetic inactivation in hematopoietic cells before leukemia onset may not necessarily recapitulate inhibition of HIF functions in overt leukemia (24). These are all interesting open questions that will need to be addressed in the future to better understand the biology of leukemia and the targetability of this pathway for leukemia treatment.

## Discussion

Hypoxia-responsive transcription factors are being increasingly implicated in the regulation of many normal and pathological cellular processes that expand well beyond mediating metabolic adaptation to oxygen deprivation, which led to their evolutionary emergence. Their increasing pervasiveness in cell biology is probably due to a number of reasons. First and foremost, their constitutive expression in conditions of physiological hypoxia. Most cell lines in use in laboratories around the world have been adapted to *in vitro* culture at pO_2_ of ambient air (160 mm Hg). However, direct measurement of oxygen levels *in vivo* reveals that much lower oxygen tensions are homeostatic in many tissues. The BM is a typical example of an organ where low oxygen levels appear directly implicated in maintenance of both normal hematopoietic and leukemic cells. This is particularly true in tissue microenvironments such as stem cell niches, where HIF factors promote a condition of metabolic “dormancy” based on a shift from mitochondrial metabolism to glycolysis that lowers production of DNA-damaging reactive oxygen species (ROS), and at the same time activate molecular programs implicated in stem cell maintenance. In addition to local hypoxia, growing evidence indicates that HIF1α and HIF2α expression is also promoted by extracellular stimuli or cell adhesion molecules provided by stromal cells, which probably reinforce HIF activity in specific BM microenvironments. As a consequence, hypoxic responses may be intrinsically wired in some hematopoietic and leukemic cells and occur at least in part independently of oxygen levels.

HIF1α and HIF2α share substantial homology and a similar structural organization, yet emerging literature is revealing diverging transcriptional outputs. The growing complexity of HIFα specific functions appears to be governed at different levels: i) tissue-specific expression of HIFα genes; ii) different sensitivity to oxygen concentrations of HIFα proteins; iii) different oxygen-independent regulation; iv) different nuclear diffusion properties and DNA binding profiles; v) binding to specific partners that promote cooperative transcriptional activation; vi) tissue-specific epigenetic modulation of target genes. The sum of all of these events leads to a diversification of HIF1α and HIF2α functions with non-overlapping consequences that are constantly emerging in physiological and pathological conditions.

With this in mind, it is to be expected that the importance of this pathway in biology will continue to grow. As per the role of hypoxia signaling in oncology, at current time a large amount of work has described the implication of HIFα factors in solid tumors, where in most cases they promote features of tumor aggressiveness, such as metastatic spread and relapse after treatment. Conversely, although hypoxia is a physiological hallmark of hematopoietic organs, the contribution of HIF1α and HIF2α to hematological malignancies has been underestimated for a long time. In recent years, many research groups have started to look into the function of hypoxia-responsive genes in blood malignancies. As we have summarized in this manuscript, important leukemia-promoting functions of HIF factors have emerged across distinct types of leukemia, including protection of LSCs, leukemia dissemination, leukemia expansion and impaired sensitivity to apoptosis and chemotherapy. Nonetheless, parallel work suggests that HIFα factors may not be essential or rather play tumor-suppressive functions in leukemia. Explanations to these contradictory results may rely on divergence of functions in distinct leukemia subtypes or at the preleukemic stage versus overt leukemia. In this respect, most of the studies that describe tumor-suppressive functions of HIFα factors in leukemia are based on genetic inactivation of these genes at a preleukemic stage, or concomitantly to leukemia initiation by mutated oncoproteins, a circumstance where it may be difficult to disentangle the function of HIFα genes in hematopoiesis or leukemia. In conclusion, further investigation is required to fully elucidate the extent and relevance of hypoxia-responsive gene activation in leukemia. In so doing, we should also aim to address the translational implications of inhibiting these pathways for the treatment of blood malignancies.

In this respect, an important note is that the vast majority of studies performed in leukemic contexts have focused on the HIF1α gene, with much less effort into elucidating the functions of HIF2α. Because HIF2α is specifically expressed in some cell types and its transcriptional output may differ from that of HIF1α, additional work may uncover specific context-dependent functions of HIF2α that are different from those exerted by HIF1α. Of relevance, because a specific small molecule inhibitor of HIF2α (belzutifan) has been recently approved by the U.S. FDA (Food and Drugs Administration) for the treatment of adult patients with VHL disease associated with renal cell carcinoma or pancreatic tumors, future studies into this pathway may pave the way for the use of this compound in other malignancies including blood cancers.

## Author contributions

Both authors collected and reviewed relevant literature and wrote this manuscript.

## Funding

This work was supported by the Italian Ministry of Health (Ricerca Finalizzata, RF-2019-12369841).

## Conflict of interest

The authors declare that the research was conducted in the absence of any commercial or financial relationships that could be construed as a potential conflict of interest.

## Publisher’s note

All claims expressed in this article are solely those of the authors and do not necessarily represent those of their affiliated organizations, or those of the publisher, the editors and the reviewers. Any product that may be evaluated in this article, or claim that may be made by its manufacturer, is not guaranteed or endorsed by the publisher.
